# Identification of a novel, MSC-induced macrophage subtype via single-cell sequencing: implications for intervertebral disc degeneration therapy

**DOI:** 10.3389/fcell.2023.1286011

**Published:** 2024-01-11

**Authors:** Jinsha Koroth, Casey Chitwood, Ramya Kumar, Wei-Han Lin, Benjamin T. Reves, Todd Boyce, Theresa M. Reineke, Arin M. Ellingson, Casey P. Johnson, Laura S. Stone, Kimberly C. Chaffin, Narendra K. Simha, Brenda M. Ogle, Elizabeth W. Bradley

**Affiliations:** ^1^ Department of Orthopedic Surgery, Medical School, University of Minnesota, Minneapolis, MN, United States; ^2^ Department of Biomedical Engineering, College of Science and Engineering, University of Minnesota, Minneapolis, MN, United States; ^3^ Department of Chemical and Biological Engineering, Colorado School of Mines, Golden, CO, United States; ^4^ Department of Chemistry, College of Science and Engineering, University of Minnesota, Minneapolis, MN, United States; ^5^ Medtronic, Memphis, TN, United States; ^6^ Stem Cell Institute, University of Minnesota, Minneapolis, MN, United States; ^7^ Department of Rehabilitation Medicine, School of Medicine, University of Minnesota, Minneapolis, MN, United States; ^8^ Department of Veterinary Clinical Sciences, College of Veterinary Medicine, University of Minnesota, St. Paul, MN, United States; ^9^ Center for Magnetic Resonance Research, University of Minnesota, Minneapolis, MN, United States; ^10^ Department of Anesthesiology, School of Medicine, University of Minnesota, Minneapolis, MN, United States; ^11^ Medtronic, Minneapolis, MN, United States

**Keywords:** STRO3, IFN gamma, single cell sequence (scRNA-seq), back pain, Coffilin, CCL2

## Abstract

Intervertebral disc (IVD) degeneration is a common pathological condition associated with low back pain. Recent evidence suggests that mesenchymal signaling cells (MSCs) promote IVD regeneration, but underlying mechanisms remain poorly defined. One postulated mechanism is via modulation of macrophage phenotypes. In this manuscript, we tested the hypothesis that MSCs produce trophic factors that alter macrophage subsets. To this end, we collected conditioned medium from human, bone marrow-derived STRO3^+^ MSCs. We then cultured human bone marrow-derived macrophages in MSC conditioned medium (CM) and performed single cell RNA-sequencing. Comparative analyses between macrophages cultured in hypoxic and normoxic MSC CM showed large overlap between macrophage subsets; however, we identified a unique hypoxic MSC CM-induced macrophage cluster. To determine if factors from MSC CM simulated effects of the anti-inflammatory cytokine IL-4, we integrated the data from macrophages cultured in hypoxic MSC CM with and without IL-4 addition. Integration of these data sets showed considerable overlap, demonstrating that hypoxic MSC CM simulates the effects of IL-4. Interestingly, macrophages cultured in normoxic MSC CM in the absence of IL-4 did not significantly contribute to the unique cluster within our comparison analyses and showed differential TGF-β signaling; thus, normoxic conditions did not approximate IL-4. In addition, TGF-β neutralization partially limited the effects of MSC CM. In conclusion, our study identified a unique macrophage subset induced by MSCs within hypoxic conditions and supports that MSCs alter macrophage phenotypes through TGF-β-dependent mechanisms.

## Introduction

Low back pain affects millions of people worldwide, with up to 80% of adults experiencing low back pain at some point in their lives (Andersson, 1999; Martin et al., 2008). Low back pain strongly associates with intervertebral disc (IVD) degeneration characterized by loss of cellularity, extracellular matrix degradation, leukocyte infiltration, and compromised structural integrity of the IVD (Vos et al., 2016). IVD degeneration exhibits a complex etiology, with factors such as age, diet, genetics, loading, and injury contributing to morbidity (Elfering et al., 2002; Livshits et al., 2011; Nasto et al., 2013). Palliative treatments help mitigate pain associated with IVD degeneration, but do not target the underlying pathological drivers of pain. Likewise, operative treatments aimed at removing or stabilizing affected IVDs offer limited efficacy in mitigating pain; therefore, understanding cellular and molecular underpinnings of the degenerative process within the IVD will provide crucial knowledge to advance treatment of this condition.

Macrophages represent a highly plastic and diverse set of cells that both promote and subsequently limit the inflammatory response during the course of tissue healing (Weivoda and Bradley, 2023). Recent evidence documents a link between local levels of inflammation within the IVD and degenerative severity (Nakazawa et al., 2018; Zhang et al., 2021; Zhao et al., 2021; Koroth et al., 2023). Specifically, macrophage marker levels positively correlate with disc degeneration in human cadaveric IVD specimens (Nakazawa et al., 2018). In contrast, mixed macrophage phenotypes (e.g., inflammatory and anti-inflammatory) are present at various stages of disc degeneration (Nakawaki et al., 2019; Lee et al., 2020; Jin et al., 2022; Yamamoto et al., 2022; Koroth et al., 2023). These data suggest that modulating the immune phenotype of macrophages in the disc may attenuate disc degeneration and/or promote regeneration of the disc (Koroth et al., 2023), but our understanding of the effects of different macrophage subsets to IVD degeneration is limited.

Mesenchymal signaling cells (MSCs, e.g., mesenchymal progenitor cells) secrete cytokines and chemokines that exert anti-inflammatory and immunomodulatory properties (Gao et al., 2016; Caplan, 2017; See et al., 2011) that make MSCs attractive candidates for the treatment of conditions such as IVD degeneration. The current prevailing route of administration for MSCs as a therapeutic agent to limit disc degeneration is via direct delivery of MSCs to the disc. Preclinical studies likewise suggest that MSCs may limit disc degeneration and/or promote regeneration (Ghosh et al., 2012; Pereira et al., 2016; Hiyama et al., 2022). One study suggested that systemic delivery of MSCs alters immune cell levels within degenerated rat discs (Cunha et al., 2017). While this study suggests that MSCs modulate immune cells in this context, it does not directly address how MSCs modify macrophage biology and/or how this is modified by changes in oxygen tension.

Early and on-going clinical trials investigating the safety and efficacy of MSCs for the treatment of IVD degeneration have yielded mixed results (Noriega et al., 2017; Amirdelfan et al., 2021; Zhang et al., 2022). Some studies reported improvements in pain and function following MSC injection into the disc; however, a better understanding of the cellular and molecular actions of MSCs on the degenerating disc would help to optimize the design of clinical trials and implementation of MSCs as IVD degeneration modifying agents.

In a therapeutic setting, delivery of MSCs into the avascular IVD imparts a change in oxygen tension (Chen et al., 2014); however, the effects of altered oxygen tension on MSC characteristics and their potential paracrine effects are often overlooked. In our current work, we investigated how trophic factors produced by MSCs alter macrophage subsets, specifically examining how oxygen tension (e.g., hypoxia versus normoxia) alters the trophic effects of MSCs. Here we report that paracrine factors produced by MSCs cultured in hypoxia (i.e., 2% O_2_) induce a unique macrophage subset as compared to factors produced by MSCs cultured at atmospheric oxygen tension. These data provide a detailed description of how trophic factors produced by MSCs affect macrophage phenotypes in the context of IVD therapy.

## Methods

### Isolation and differentiation of macrophages from human bone marrow

Human whole bone marrow aspirates from female, non-smokers aged 18–35 years old were procured from Lonza Corporation. Bone marrow aspirates were diluted 6X with RBC Lysis buffer (eBioscience, #00-4333-57) followed by a 5 min incubation at room temperature. Nucleated cells from whole bone marrow were pelleted at 1,000 rpm for 5 min, and the supernatant was removed and discarded. The cell pellet was suspended in MACS buffer (phosphate-buffered saline (PBS), pH 7.2, 0.5% bovine serum albumin (BSA), and 2 mM ethylene-diamine-tetra acetic acid (EDTA) prepared by diluting Miltenyi Biotech MACS BSA Stock Solution, # 130-091-376 1:20 with Miltenyi Biotech autoMACS Rinsing Solution, # 130-091-222) and filtered through 70 μm followed by 35 μm cell strainers. A subset of nucleated cells (5 × 10^6^ cells) were reserved for MACS validation via flow cytometry (*n* = 3 donors). Cells were counted and pelleted at 1,000 rpm. Nucleated cells within the pellet were suspended in 100 μL 1X MACS Buffer per 1 × 10^7^. Cells were then incubated with Allophycocyanin (APC)-conjugated anti-Cluster of differentiation (CD)14 (10 μL per 1 × 10^7^ cells) for 10 min at 4C. Cells were pelleted, washed, suspended in 1X MACS buffer (80 μL per 1 × 10^7^ cells) and incubated with anti-APC microbeads (20 μL per 1 × 10^7^ cells) for 15 min at 4C. Cells were washed, suspended in 500 μL MACS buffer. An LS MACS separation column (Miltenyi Biotech, #130-042-401) was placed within the magnetic field of a MACS Separator magnet and the column was equilibrated with 3 mL 1X MACS buffer. Following equilibration, anti-APC microbead-labeled cells were applied to the LS column. The column was washed 3X with 3 mL 1X MACS buffer. To isolate APC-labeled cells, the column was removed from the magnet and cells were flushed from the column with 5 mL 1X MACS buffer. Eluted cells were then pelleted at 1,000 rpm for 5 min, and the supernatant was removed and discarded. APC-labeled cells were then suspended in 100 μL 1X MACS Buffer per 1 × 10^7^ cells and incubated with phycoerythrin (PE)-conjugated anti-CD11B (10 μL per 1 × 10^7^ cells) for 10 min at 4C. Cells were washed, suspended in 1X MACS buffer (80 μL per 1 × 10^7^ cells), and incubated with anti-PE microbeads (20 μL per 1 × 10^7^ cells) for 15 min at 4C. Magnetic sorting for PE-labeled cells was accomplished as described above. To validate effective sorting of CD14^+^/CD11B^+^ cells via MACS, flow cytometry was performed (*n* = 3). For flow cytometry analysis, we prepared single stained controls and double stained samples with unsorted nucleated cells with PE-conjugated anti-CD11B, APC-conjugated anti-CD14, or co-stained with anti-CD11B and anti-CD14 antibodies (1 × 10^6^ cells per condition), respectively. Data were acquired on a BD LSR II (H1010) and analyses were performed using BD FACSDiva^TM^ software.

### Culture of human bone marrow-derived macrophages

Macrophages were generated from MACS sorted cells by culturing isolated CD14^+^/CD11B^+^ cells at a density of 0.26 × 10^6^ per cm^2^ in α-Minimal Essential Medium supplemented with 50 ng/mL recombinant human macrophage-colony stimulating factor (M-CSF) (R&D Systems, 416-ML) at atmospheric oxygen tension at 37C. Cells were maintained in culture and supplemented with M-CSF for 7 days or until adherent. To assess survival time in hypoxia, we expanded cells to 80% confluence, and then transferred to 2% oxygen tension at 37C. Cells were then fixed and TUNEL stained (Sigma-Millipore, #11684795910) to determine the percent terminal deoxynucleotidyl transferase dUTP nick end labeling (TUNEL) positive cells as a measure of apoptosis (*n* = 3). To validate macrophage responsiveness, isolated CD14^+^/CD11B^+^ cells were treated with 20 ng/mL Interferon (IFN)-γ or 100 ng/mL Interleukin (IL)-4 for 30 min or 24 h.

### Western blotting

Cell lysates were collected in a buffered *sodium dodecyl sulfate (*SDS) solution (0.1% glycerol, 0.01% SDS, 0.1 m Tris, pH 6.8) on ice. Total protein concentrations were obtained with the Bio-Rad D_C_ assay (Bio-Rad). Proteins (20 μg) were then resolved by SDS-polyacrylamide gel electrophoresis (PAGE) and transferred to a polyvinylidene difluoride membrane. Western blotting was performed with antibodies (1:2000 dilution) for phospho-S727 Signal Transducer and Activation of Transcription (STAT)1 (Cell Signaling Technology, #8826), STAT1 (Cell Signaling Technology, #9172), phospho-STAT6 (Cell Signaling Technology, #9361), STAT6 (Cell Signaling Technologies, #9362), Histone 3 (Abcam, #ab1791) and corresponding secondary antibodies conjugated to horseradish peroxidase (HRP) (Cell Signaling Technology). Antibody binding was detected with the Supersignal West Femto Chemiluminescent Substrate (Thermo Fisher Scientific, #34096). Western blotting was repeated in three independent replicate experiments, each containing pooled replicate wells per group.

### RNA isolation and qRT-PCR

Total RNA was extracted from human macrophages using TRIzol (Thermo Fisher, #15596018) and chloroform, and 1 μg was reverse transcribed using the iScript™ Reverse Transcription Supermix (Biorad, #1708841). The resulting cDNAs were used to assay gene expression via real-time PCR using the gene-specific primers listed in Table 1. Fold changes in gene expression for each sample were calculated using the 2^−ΔΔCT^ method relative to control after normalization of gene-specific C_t_ values to GAPDH C_t_ values (Mattson et al., 2019; Molstad et al., 2020). Shown are data from three independent replicate experiments.

**TABLE 1:** Primer Sequences use for qPCR.

Gene	Forward Primer Sequence	Reverse Primer Sequence
GAPDH	5′-TCG​GAG​TCA​ACG​GAT​TTG​GT-3′	5′-TTC​CCG​TTC​TCA​GCC​TTG​AC-3′
MRC1	5′-GTG​ATG​GGA​CCC​CTG​TAA​CG-3′	5′-CTG​CCC​AGT​ACC​CAT​CCT​TG-3′
PPARG	5′-CCA​GAA​GCC​TGC​ATT​TCT​GC-3′	5′-GTG​TCA​ACC​ATG​GTC​ATT​TCG​TT-3′
IL-1β	5′-TTC​GAG​GCA​CAA​GGC​ACA​A-3′	5′-TGG​CTG​CTT​CAG​ACA​CTT​GAG-3′
IL-1RA	5′-GCC​TCC​GCA​GTC​ACC​TAA​T-3′	5′-TTA​ACA​TCC​CAG​ATT​CTG​AAG​GC-3′
IL-6	5′-CAT​CCT​CGA​CGG​CAT​CTC​AG-3′	5′-ACC​AGG​CAA​CTC​TCC​TCA​TTG-3′
TNF-α	5′-TGC​ACT​TTG​GAG​TGA​TCG​GC-3′	5′-CTC​AGC​TTG​AGG​GTT​TGC​TAC-3′

### Generation of MSC conditioned media

Isolation, expansion and characterization of human MSCs from whole bone marrow of a Caucasian male 18–35 years old was previously described (Jung et al., 2016). MSCs were cultured at a density of 5,000 cells/cm^2^ for 24 h in either atmospheric oxygen tension or 2% O_2_. Conditioned medium from cultured MSCs was then collected and stored at −80C for future use. To block effects of TGF-β isoforms, MSC conditioned media were incubated with the TGF-β isoform neutralizing antibody 1D11 (30 µg/mL, Thermo Fisher, #MA523795) for 2 h prior to use.

### Preparation of macrophages for scRNA-Seq

CD14^+^/CD11B^+^ cells were isolated via magnetic-assisted cell sorting (MACS) and we generated human macrophages as described above. We then stimulated macrophages with 10 ng/mL IFN-γ and cultured cells at 2% O_2_ for 24 h. IFN-γ-containing medium was then replaced with MSC-conditioned media as detailed below and macrophages were maintained at 2% O_2_ for 24 h in MSC-conditioned media. Cells were then rinsed with PBS and incubated Macrophage Detachment Solution (Sigma Millipore, #41330) for 35 min to remove cells from plastic. Cells were counted and pelleted at 1,000 rpm for 5 min. Dead cells were cleared from the cell suspension utilizing the Dead Cell Removal Kit (Miltenyi, #130-090-101) as per the manufacturer’s instructions. Macrophages were suspended at a density of 700–1,200 cells/µL in MEM with 10% FBS. Cell viability was assessed using a Luna Cell Counter to ensure greater than 75% viability before proceeding to scRNA-Seq.

### scRNA-seq library construction

The libraries were prepared with Chromium Single cell 3′ Reagent v3 Kits (10× Genomics, Pleasanton, CA) according to the manufacturer’s protocol. Each single-cell suspension was mixed with primers, enzymes, and the gel beads containing barcode information, and then loaded on a Chromium Single Cell Controller (10× Genomics) to generate single-cell gel beads in emulsions (GEMs). Each gel bead was bonded to one cell and encased within oil surfactant. After generating the GEMs, reverse transcription was performed using barcoded full-length cDNA followed by the disruption of emulsions using the recovery agent and cDNA clean up with DynaBeads MyOne Silane Beads (Thermo Fisher Scientific). cDNA was then amplified by PCR with an appropriate number of cycles and thermal conditions that depended on the recovery cells. Subsequently, the amplified cDNA was fragmented, end-repaired, A-tailed, ligated to an index adaptor, and subjected to library amplification. Quality of amplified cDNA was assessed using an Agilent Bioanalyzer prior to high-throughput sequencing. Following quality control, the cDNA libraries were sequenced on an Illumina NovaSeq 6000 sequencer. The Genomics Core at the University of Minnesota performed library construction and sequencing.

### Single cell bioinformatic analyses

**FIGURE 1:**
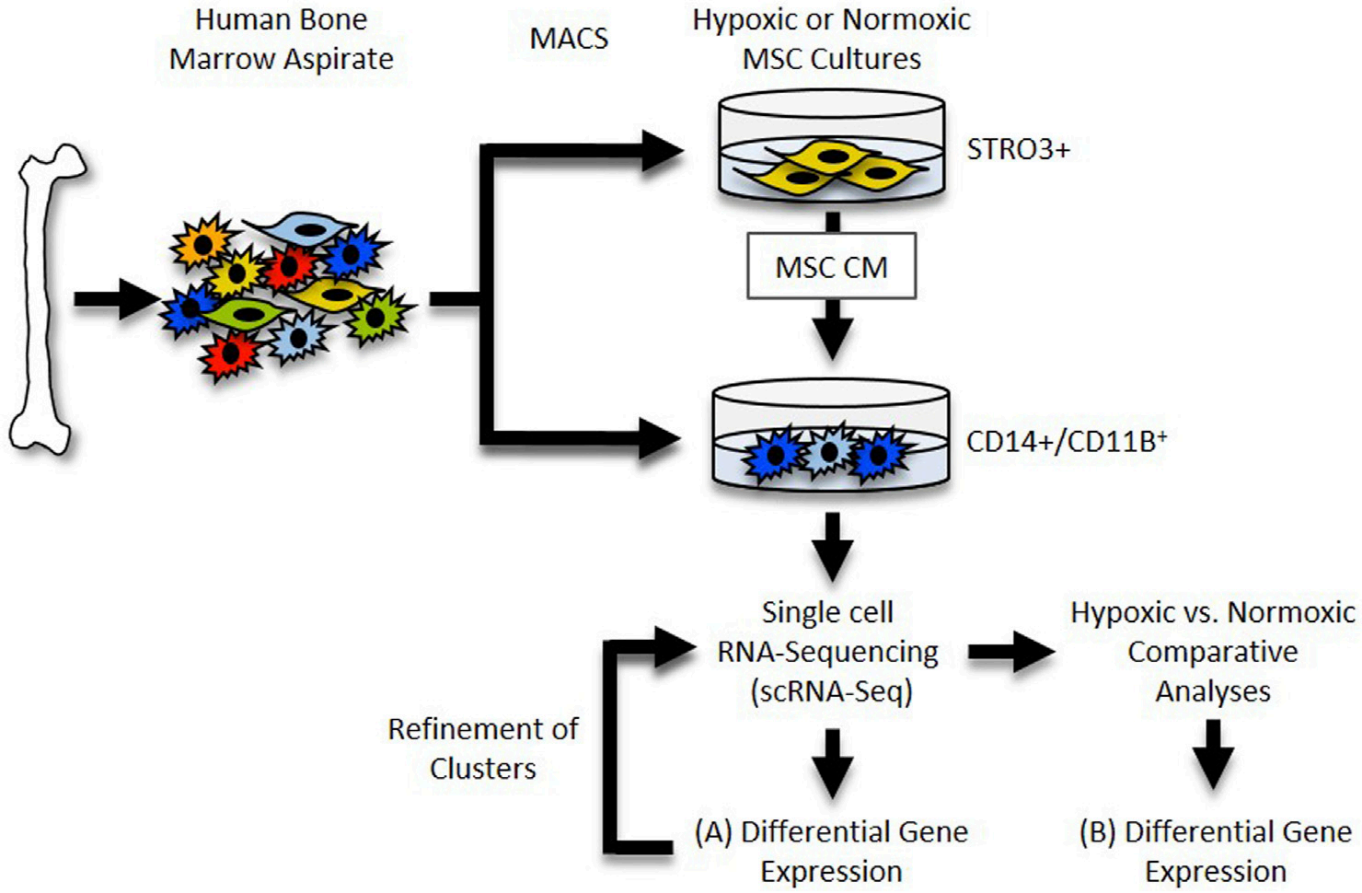
Single cell RNA-Seq Workflow. Diagram depicting generation and analysis of scRNA-Seq datasets. **(A)** Datasets for each sample were generated and differential gene expression within each cluster was determined. **(B)** Datasets were compared and differential gene expression within clusters between samples was determined.

The 10× Genomics Cell Ranger software (version 6.0.0) was used to process the raw scRNA-seq data in accordance with the guidelines specified by the manufacturer. Cell Ranger software (2020-A, 10× Genomics Cell Ranger Count v7.0.1) was used to demultiplex the raw base call (BCL) files produced by the Illumina NovaSeq 6000 sequencer. Next, Cell Ranger was used to count the unique molecular identifiers (UMI) and barcodes and align fastq files to the human reference genome (GRCh38). We then used R software (4.2.2) with the Seurat package (4.3.0) (Satija et al., 2015) to conduct quality assurance and subsequent studies on the feature-barcode matrices created by Cell Ranger. The study was filtered to exclude cells with outlier status, aberrant gene detection rates (<500 and >5,000), and high mitochondrial transcript level (>8%), a sign of cellular stress. Possible doublets were also excluded from the data using doubletFinder for subsequent analysis. The remaining cells were then normalized, and a set of 2000 highly variable genes were selected based on a variance stabilizing transformation (“vst”). Next, the data was scaled to account for technical variability in the data and used for principal component analysis (PCA) to reduce the dimensionality of the data. Using Seurat’s “FindNeighbors” and “FindClusters” functions, cells were then organized into an ideal number of clusters for *de novo* cell type discovery. Then, using uniform manifold approximation and projection (UMAP), a non-linear dimensional reduction was carried out, enabling for the identification and visualization of different cell clusters. Then, clusters over expressing “TAGLN,” “SERPINE1,” “TPM1,” “FN1” and “IGFBP7” genes (e.g., MSC contaminants) were removed, and the remaining data were normalized. After variable feature identification, the data were used for integrated analysis. The “VlnPlot” and “FeaturePlot” functions of Seurat were used to construct gene expression plots. For an overview of this analysis pipeline, See Figure 1.

### Comparison of CD11D-CD14 cells from different conditioned media

The data from different conditioned media/treatments were integrated for downstream analysis. Seurat’s integration pipeline (Butler et al., 2018) involves aligning the low-dimensional representations of each dataset using canonical correlation analysis (CCA). CCA identifies the common sources of variation between datasets and projects them onto a shared embedding, allowing for the identification of cell types and states that are shared across multiple datasets (Butler et al., 2018; Rodda et al., 2018). “FindIntegrationAnchors” method was used to find anchors for data integration. Then, “IntegrateData” function was used with these anchors to create a new integrated matrix using selected datasets. Dimensionality reduction, grouping, and visualization processes were then carried out in Seurat as described above. Using the “FindMarkers” function, genes with differential expression between two conditions were identified.

## Results

### Development and validation of a method for generation of human macrophages from whole bone marrow via magnetic assisted cell sorting (MACS)

**FIGURE 2:**
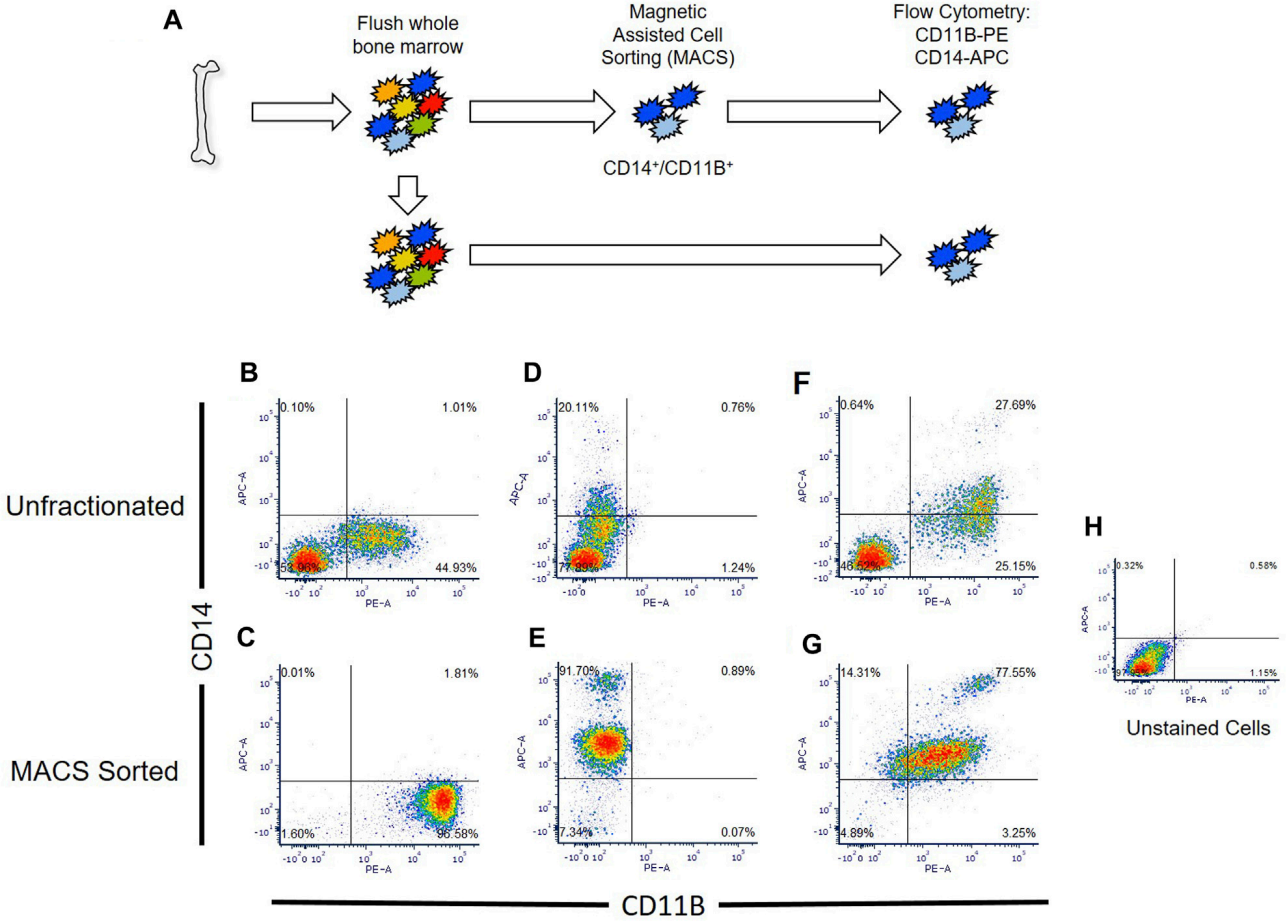
Comparison of flow cytometry sorted and Magnetic Assisted Cell Sorting (MACS) isolated CD14^+^/CD11B^+^ cells. **(A)** Overview of analyses. Human bone marrow aspirates were collected and sorted via flow cytometry or MACS. **(B–G)** Unfractionated nucleated bone marrow cells (upper panels) or MACS sorted cells (lower panels) were stained with anti-CD14 **(B,C)**, anti-CD11B **(D,E)**, or co-stained with anti-CD11B and anti-CD14 antibodies **(F,G)**. Flow cytometry was then performed to detect single and double stained cells in each sample. Unstained nucleated bone marrow cells were used as a negative control **(H)**.

Primary macrophages easily lyse during conventional cell sorting (e.g., flow cytometry, FACS), generating macrophage cellular remnants that can bind to other cell types and induce artifacts within datasets (Millard et al., 2021). To circumvent this limitation, we developed a protocol for isolation and generation of human macrophages utilizing sequential magnetic assisted cell sorting (MACS) for CD14^+^/CD11B^+^ cells (Figure 2A).

We confirmed that MACS sorted cells were CD14/CD11B double-positive cells via flow cytometry, and showed that MACS sorting yielded nearly three times as many CD14^+^/CD11B^+^ cells (e.g., 77.5% versus 27.7%) as compared to conventional flow cytometry (Figure 2B). MACS sorted CD14^+^/CD11B^+^ exhibited known bifurcation in CD14 levels, producing both CD14^low^ and CD14^high^ cells as described within the literature (Weivoda and Bradley, 2023); however, fewer CD14^high^ cells were observed via flow cytometry (Figure 2B). Using this approach, from a starting volume of 10 mL whole bone marrow aspirate, 5% ± 0.92% of nucleated cells were CD14^+^/CD11B^+^ cells. Isolation of double positive cells via MACS sorting depended on sequential isolation of CD14^+^ cells followed by isolation of CD11B^+^ cells.

**FIGURE 3:**
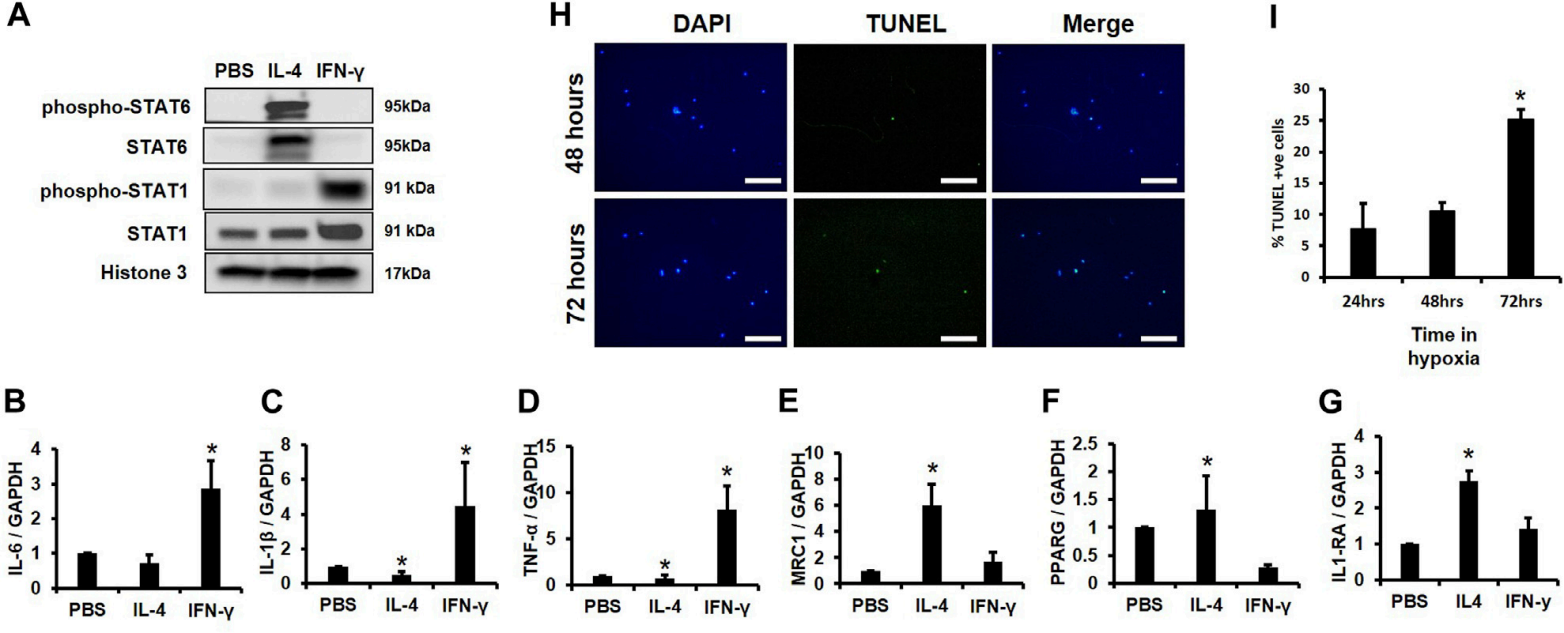
Survival Time of CD14^+^/CD11B^+^ cells Cultured in Hypoxia and Validation of Macrophage Function. **(A)** MACS sorted CD14^+^/CD11B^+^ cells were treated with cytokines for 2 h and Western blotting was performed. **(B–G)** MACS sorted CD14^+^/CD11B^+^ cells were treated with cytokines for 24 h and expression of **(B)** IL-6, **(C)** IL-1β, **(D)** TNF-α, **(E)** MRC1, **(F)** PPARG, and **(G)** IL-1RA was evaluated by qPCR *n* = 3, *p < 0.05. **(H,I)** MACS sorted CD14^+^/CD11B^+^ cells were cultured in 2% oxygen for 24, 48, or 72 h. Cells were then fixed and **(H)** TUNEL and DAPI stained and the **(I)** percent TUNEL^+^ cells was evaluated, *n* = 3,*p < 0.05. Scale bars in H are 100 microns.

For subsequent experiments, we needed to demonstrate that our MACS isolated cells could functionally respond to pro- and anti-inflammatory cytokines. We first determined if our MACS-sorted cells responded to IL-4, an anti-inflammatory cytokine previously shown to induce STAT6 activation, reduce expression of inflammatory mediators, and promote IL-1RA production (Nelms et al., 1999). IL-4 induced phosphorylation and activation of STAT6 (Takeda et al., 1996), but not STAT1 (Figure 3A) (Weivoda and Bradley, 2023). In contrast, pro-inflammatory IFN-γ stimulated STAT1 phosphorylation and activation, but did not alter STAT6 activity (Figure 3A). Furthermore, expression of the inflammatory markers IL-6, IL-1β, and TNF-α increased following IFN-γ addition (Figures 3B–D), but IFN-γ-stimulated cells did not produce changes in levels of the macrophage markers MRC1, PPARG, and IL-1RA (Figures 3E–G). In addition, we also observed enhanced expression of the macrophage marker genes MRC1, PPARG, and IL-1RA following IL-4 addition (Figures 3E–G), whereas IL-4 suppressed expression of the pro-inflammatory markers IL-1β and TNF-α as compared to controls as previously reported (Figures 3B–D) (Hart et al., 1989).

Next, we assessed survival time in hypoxia (i.e., 2% O_2_) of MACS isolated CD14^+^/CD11B^+^ cells. No significant changes in TUNEL staining were observed following 24 or 48 h in hypoxic conditions, but an approximate 15% increase in TUNEL positive cells was observed after 72 h (Figures 3H, I). These data support that MACS isolated macrophages effectively respond to cytokine exposure and demonstrate sufficient survival in hypoxia at 24 or 48 h; thus, as 24-hour time point was used for subsequent experiments.

### Isolation of STRO3^+^ MSCs and generation of conditioned medium

**FIGURE 4:**
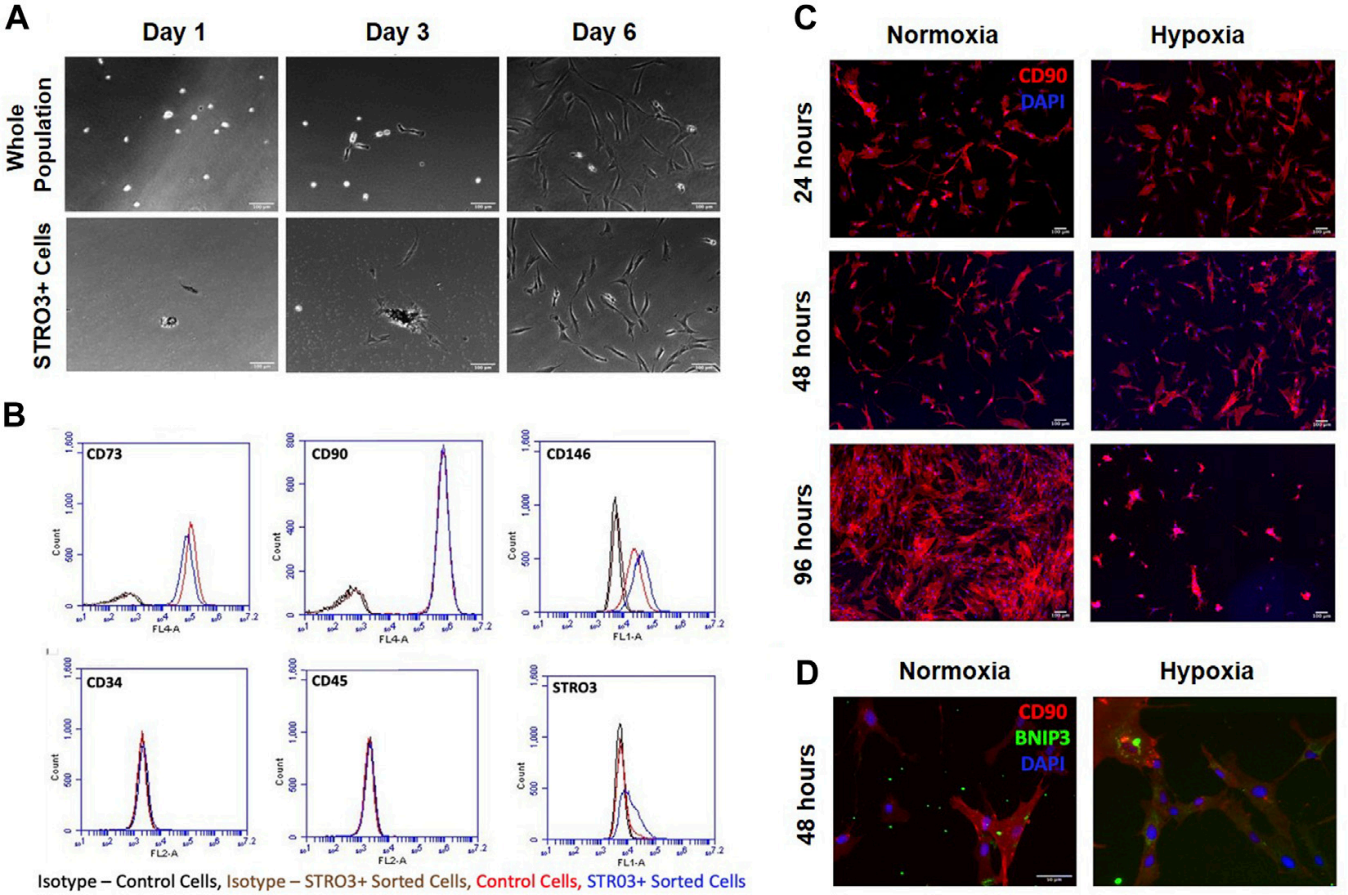
MSC Isolation and Survival in Hypoxia. MSCs were isolated from human bone marrow aspirates based on plastic adherence of whole bone marrow or expression of the STRO3 antigen. **(A)** Images from the first 6 days of culture post isolation for both the whole population of MSCs and STRO3^+^ MSCs. **(B)** Flow cytometry analysis of whole bone marrow and STRO3^+^ MSCs. **(C)** STRO3^+^ MSCs were cultured in hypoxia as shown and immunostained for the MSC surface marker Cd90 and DAPI stained. **(D)** STRO3+ MSCs were cultured in hypoxia for 48 h and immunostaining for Cd90 and BNIP3 was performed.

Prior studies report that the STRO3 antigen marks MSCs that exhibit greater regenerative properties (See et al., 2011; Ghosh et al., 2012). To determine how trophic factors produced by MSCs affect macrophage phenotypes, we isolated MSCs from human bone marrow aspirates based on plastic adherence or expression of the STRO3 antigen via MACS (Gronthos et al., 2007; Oehme et al., 2016; Abdalmula et al., 2017) (Figure 4A). We confirmed purity of MSCs via flow cytometry for CD73, CD90, CD146, CD34, CD45 and STRO3 (Dominici et al., 2006; Ma et al., 2021). Our isolated MSCs were greater than 95% positive for CD73 and CD90, and greater than 45% positive for CD146 (Figure 4B). Furthermore, enrichment for STRO3^+^ cells did not significantly affect MSC purity when compared to MSCs isolated via plastic adherence (Figures 4A, B). To generate conditioned medium, we cultured STRO3^+^ enriched MSCs for 24 h at either atmospheric oxygen tension, or 2% O_2_ to simulate the hypoxic environment of the intervertebral disc. MSCs cultured in hypoxia exhibited reduced survival time, as evidenced by reduced numbers of CD90^+^ cells (Figure 4C) and enhanced expression of BNIP3, a marker of cell death induced by hypoxia (Zhang and Ney, 2009; Deng et al., 2022), after 48 h (Figure 4D). To limit the confounding effects of cell death in hypoxia, MSCs were cultured for 24 h in either normoxic or hypoxic conditions and CM were collected for subsequent experiments.

### Effects of normoxic MSC conditioned medium on macrophage subsets

**FIGURE 5:**
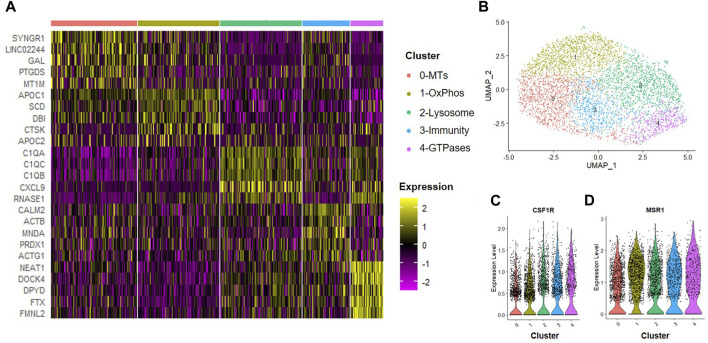
Macrophage Subsets Present Following Exposure to Normoxic MSC Conditioned Medium. Human macrophages were cultured in normoxic MSC conditioned medium for 24 h scRNA-Seq was performed and macrophage subsets were identified through cluster analyses. **(A)** Heatmap of top five differentially regulated genes within each cluster. **(B)** UMAP representation of cell clustering. Violin plots for expression of **(C)** CSF1R and **(D)** MSR1 by individual cells within each cluster.

Next, we assessed how trophic factors produced by MSCs maintained in normoxic conditions influenced macrophage subsets. To simulate the inflammatory environment of a degenerative disc, CD14^+^/CD11B^+^ cells were isolated from human bone marrow aspirates as described above and then cultured at 2% O_2_ in the presence of IFN-γ for 24 h to induce an inflammatory phenotype. Following induction of inflammation, we switched cells to MSC CM for 24 h. Macrophages were then isolated for scRNA-Seq analysis. We first evaluated the effects of trophic factors produced by normoxic cultured MSCs on macrophage subsets. We identified five unique macrophage clusters under these conditions (Figures 5A, B). Because we are employing a novel macrophage isolation method, we confirmed expression of the macrophage markers CSF1R (i.e., macrophage-colony stimulating factor 1 receptor) and MSR1 (i.e., macrophage scavenger receptor 1) within each cluster (Figures 5C, D) and lack of MSC-marker genes (e.g., TAGLN, FN1). To assign cluster names to macrophage subsets within each sample, we performed bioinformatic analyses using GO and DAVID software to identify functional gene annotation categories present within each cluster. Types of macrophage clusters identified included macrophages enriched for functional gene categories including 0) metallothioneins, 1) oxidative phosphorylation, 2) lysosome and antigen presentation, 3) innate immunity, and 4) GTPase signaling.

### Macrophage subsets induced by MSC secreted factors in hypoxic conditions

**FIGURE 6:**
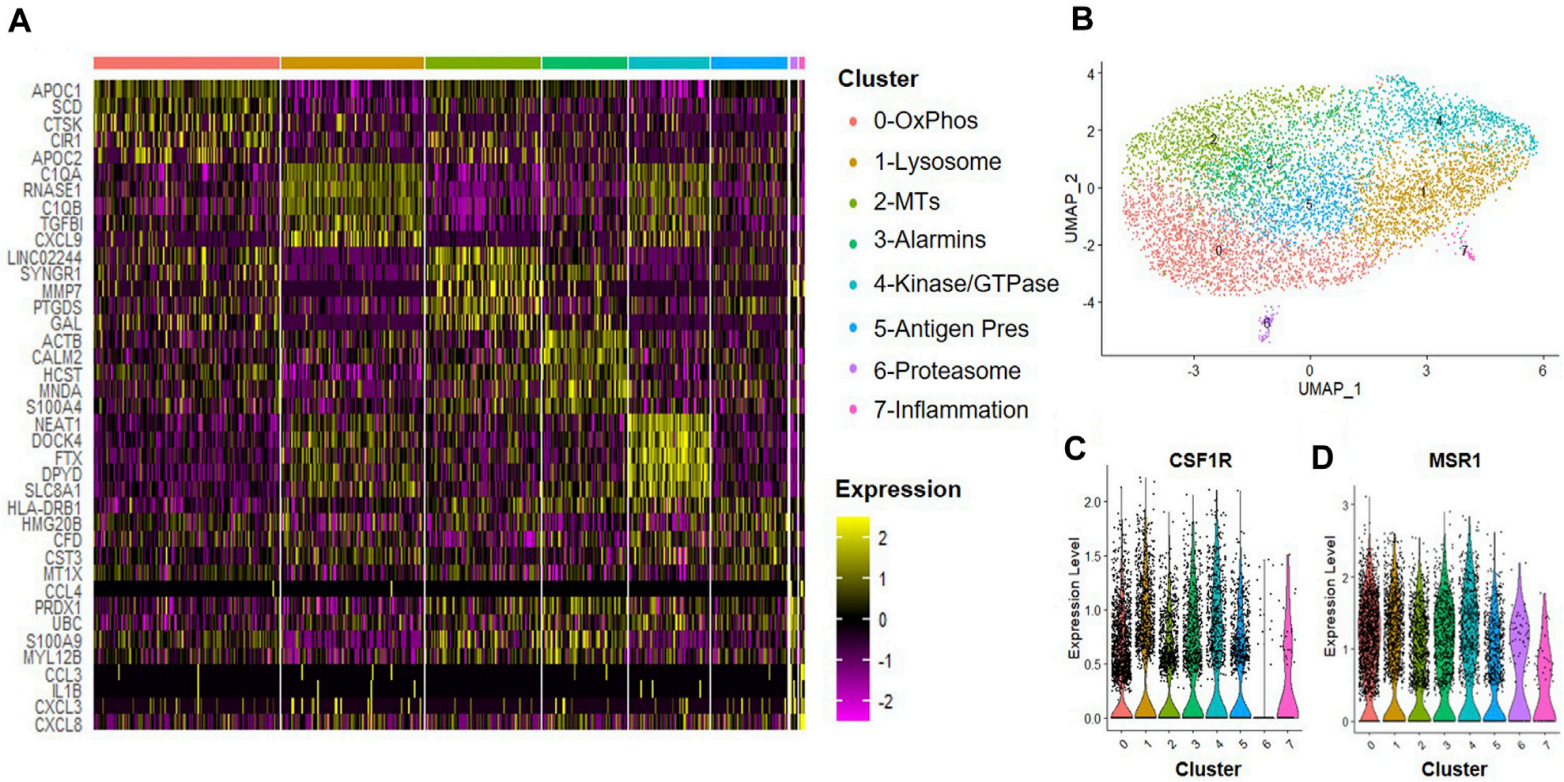
Macrophage Subsets Present Following Exposure to Hypoxic MSC Conditioned Medium. Human macrophages were cultured in hypoxic MSC conditioned medium for 24 h scRNA-Seq was performed and macrophage subsets were identified through cluster analyses. **(A)** Heatmap of top five differentially regulated genes within each cluster. **(B)** UMAP representation of cell clustering. Violin plots for expression of **(C)** CSF1R and **(D)** MSR1 by individual cells within each cluster.

A hypoxic environment characterizes the intervertebral disc; thus, MSCs delivered to this environment may produce alternate trophic factors and exert differential effects on the tissue regeneration processes, including effects on macrophages within the disc. We next evaluated the effects of trophic factors produced by hypoxic cultured MSCs on macrophage subsets. CD14^+^/CD11B^+^ cells were isolated from human bone marrow aspirates as described above and then cultured at 2% O_2_ in the presence of IFN-γ for 24 h to simulate an inflammatory environment. Cells were then switched to hypoxic MSC CM for 24 h and we performed scRNA-Seq analyses. We identified eight clusters of macrophages (Figures 6A, B) and confirmed expression of macrophage phenotypic genes within each cluster (e.g., CSF1R, MSR1, Figures 6C, D). Enriched expression of 0) oxidative phosphorylation, 1) lysosomal, 2) metallothioneins, 3) alarmins, 4) protein kinase and GTPase signaling, 5) antigen presentation, 6) proteasome, and 7) inflammatory mediator signaling genes defined clusters within this sample. Clusters 6 and 7 exhibited the most distinction from other macrophage subsets within this sample. Genes with the highest enrichment and most significant expression within cluster 6 include UBC (ubiquitin C), PRDX11 (peroxiredoxin 1), S100A9 (S100 calcium-binding protein A9), and MYL12B2 (myosin light chain 12B) (Figure 6A). Genes with the highest enrichment within cluster 7 include CCL3 (C-C motif chemokine ligand 3), IL1B, CXCL(C-X-C motif chemokine ligand) 3, and CXCL8 (Figure 6A).

### IL-4-induced macrophage subsets

**FIGURE 7:**
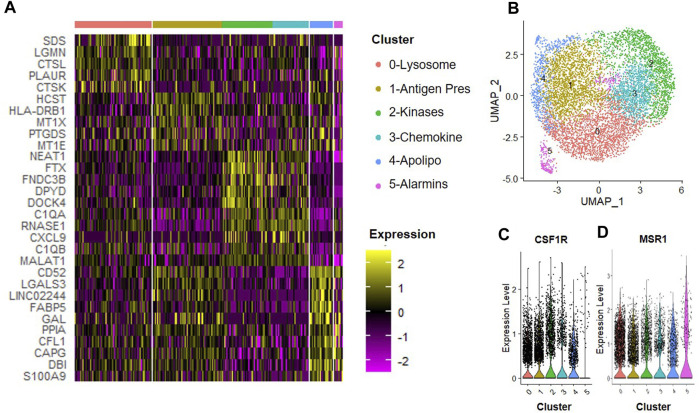
Macrophage Subsets Present Following Exposure to IL-4. Human macrophages were cultured in hypoxic MSC conditioned medium for 24 h scRNA-Seq was performed, and macrophage subsets were identified through cluster analyses. **(A)** Heatmap of top five differentially regulated genes within each cluster. **(B)** UMAP representation of cell clustering. Violin plots for expression of **(C)** CSF1R and **(D)** MSR1 by individual cells within each cluster.

We next determined how the anti-inflammatory cytokine IL-4 affected subsets of macrophages. CD14^+^/CD11B^+^ cells were isolated from human bone marrow aspirates as described above and then cultured at 2% O_2_ in the presence of IFN-γ for 24 h. We observed six unique clusters of macrophages when cells were subsequently cultured in hypoxic MSC CM supplemented with IL-4 for 24 h (Figure 7). We first confirmed that each of these clusters were macrophages based on expression of CSF1R and MSR1 (Figures 7C, D). A heatmap of the top five statically significant differentially expressed genes within each cluster is shown in Figure 7A. Types of macrophage clusters identified included macrophages enriched for expression of 0) lysosomal function, 1) antigen presentation, 2) receptor kinase signaling, 3) complement/chemokines, 4) apolipoproteins and oxidative phosphorylation, and 5) alarmins. Cluster 5 exhibited the most distinction and is marked by expression of alarmins and the surface antigens Cd81 and Cd151; thus, these surface markers could be used to specifically isolate and define the roles of this macrophage subset to disc degeneration. Genes with the highest enrichment in this cluster include S100A9 (S100 calcium binding protein A9), DBI (diazepam binding inhibitor, acyl-CoA binding protein), CAPG (capping actin protein, gelsolin like), and CFL1 (cofilin 1). Additionally, genes most significantly differentially expressed (e.g., highest *p*-value) within cluster 4 include the thymosins TMSB10 (thymosin beta 10) and TMSB4X (thymosin beta 4 X-linked).

### Hypoxia-induced MSC factors elicit a unique macrophage subset

**FIGURE 8:**
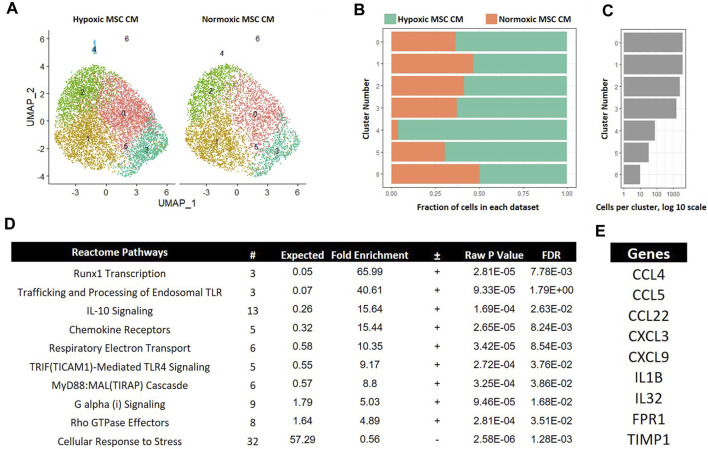
Comparative Analysis between Macrophages Cultured in Hypoxic versus Normoxic MSC Conditioned Medium. scRNA-Seq datasets from macrophages cultured in hypoxic or normoxic MSC conditioned medium were integrated and comparative analyses were performed. **(A)** UMAP representation. The fraction **(B)** and **(C)** cells from each sample within each cluster are shown. **(D)** Differentially expressed genes within cluster 4 were identified and functional categories were identified using GO analyses. **(E)** Top candidate genes were identified.

MSCs encounter a drastic decline in oxygen tension when injected into the avascular intervertebral disc (Holm and Selstam, 1982). Degeneration of the disc may likewise alter oxygen tension via recruitment of vasculature (Holm and Selstam, 1982); thus, understanding how MSCs exposed to different oxygen tensions affects macrophage subsets may offer clues as to how MSCs act therapeutically in different states of tissue degeneration. Moreover, how MSCs affect subsets of macrophages is not known. To gain insight into how MSCs exposed to different oxygen tension environments affect macrophage subsets, we first performed comparative analyses between macrophages cultured in hypoxic and normoxic MSC CM. Integration of these two data sets showed large overlap between most macrophage subsets; however, cluster four was a notable exception. Cluster 4 marked a unique macrophage subset present only when macrophages were cultured in hypoxic MSC CM (Figures 8A–C). Cluster 4 represented approximately 4% of cells within this sample (Figure 8B). Gene Ontogeny (GO) analyses using differentially expressed genes from cluster 4 of our hypoxic to normoxic comparison analysis revealed enrichment in several Reactome Pathways, including IL-10 signaling, Chemokine Receptors, G alpha (i) Signaling, and Rho GTPase Effectors (Figure 8D). Genes from these enriched pathways are listed in Figure 8E.

### Trophic factors produced by MSCs cultured in hypoxia approximate the effects of IL-4 on macrophage subsets

**FIGURE 9:**
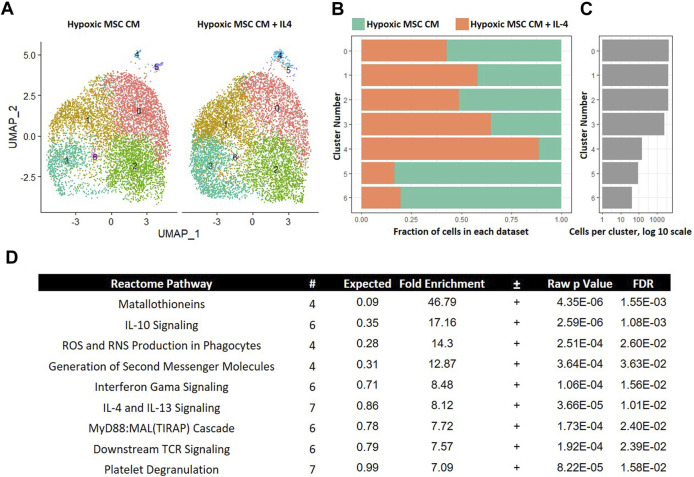
Comparative Analysis between Macrophages Cultured in Hypoxic Medium in the Presence and Absence of IL-4. scRNA-Seq datasets from macrophages cultured in hypoxic MSC conditioned medium were integrated with macrophages culture with the addition of IL-4 and comparative analyses were performed. **(A)** UMAP representation. The fraction **(B)** and **(C)** cells from each sample within each cluster are shown. **(D)** Differentially expressed genes within cluster 4 were identified and functional categories were identified using GO analyses.

Inflammation characterizes the IVD degenerative process; thus, limiting inflammation may lessen disease progression. IL-4 is a known anti-inflammatory cytokine that may lessen IVD progression (Kedong et al., 2020). We next determined if IL-4 addition altered the effects of trophic factors produced by MSCs cultured in hypoxia. To this end, we integrated data from macrophages cultured in hypoxic MSC CM with and without IL-4 addition. UMAP representations of each sample demonstrated a similar distribution of cells within clusers 0–2 (Figures 9A–C). Clusters 3 and 4 exhibited greater representation by macrophages cultured in the presence of IL-4, whereas clusters 5 and 6 were overrepresented by macrophages cultured in the absence of IL-4 (Figures 9A–C). Macrophages within cluster 5 exhibited the largest change in gene expression, with both macrophages cultured in the presence and absence of IL-4 contributing to this macrophage subset (Figures 9A–C). We assessed differentially expressed genes within cluster five of this comparison via GO analysis and noted enrichment in IL-10 Signaling, IFN-γ Signaling, and IL-4/IL-13 Signaling Reactome Pathways (Figure 9D). Differential expression of genes within the IFN-γ Signaling, and IL-4/IL-13 Signaling was expected as these two cell populations were treated with these cytokines, respectively, and support the validity of our analyses.

### MSCs cultured in normoxia do not produce factors that approximate IL-4

**FIGURE 10:**
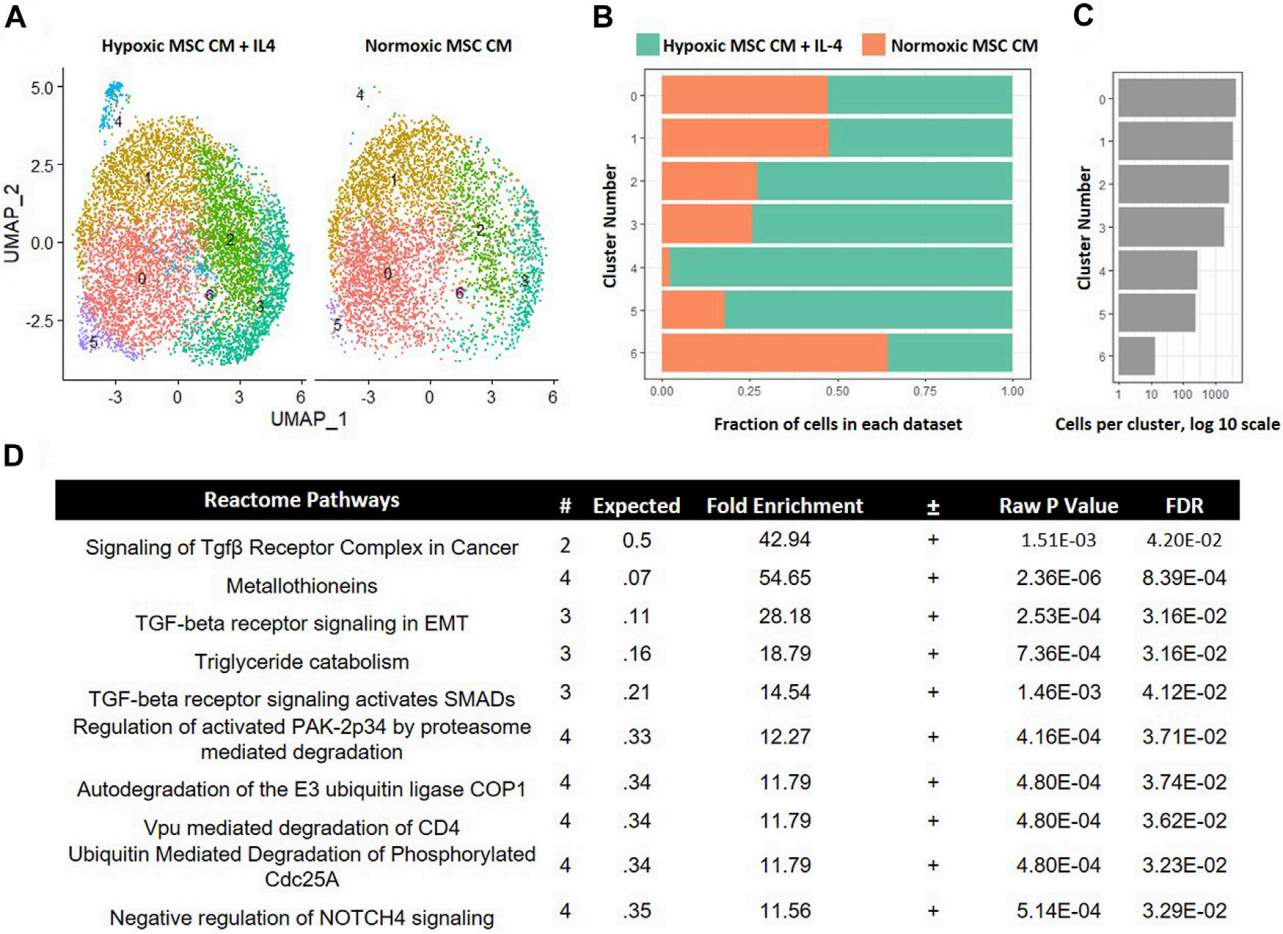
Comparative Analysis between Macrophages Cultured in Hypoxic MSC Conditioned Medium and IL-4 with Macrophages Cultured in Normoxic MSC Conditioned Medium. scRNA-Seq datasets from macrophages cultured in hypoxic MSC conditioned medium and IL-4 were integrated with macrophages cultured in normoxic MSC conditioned medium. **(A)** UMAP representation. The fraction **(B,C)** cells from each sample within each cluster are shown. **(D)** Differentially expressed genes within cluster 4 were identified and functional categories were identified using GO analyses.

In our comparison analyses, we observed that hypoxic CM induced a unique macrophage subset as compared to normoxic CM. In contrast, we did not observe unique macrophage subsets when comparing the effects of hypoxic CM in the absence and presence of IL-4. These data imply that tropic factors produced by MSCs cultured in hypoxia, but not normoxia, induce a unique macrophage subset approximating the effects of IL-4. To test further this hypothesis, we integrated the data from macrophages cultured in hypoxic MSC CM with IL-4 addition and normoxic MSC CM. Integration of these two data sets showed considerable overlap between the two samples. Interestingly, macrophages cultured in normoxic MSC CM did not significantly comprise cluster 4 within our comparison analyses (Figures 10A–C). These data suggest that normoxic conditions did not approximate the effects of IL-4 on macrophage subsets. GO analyses of differentially expressed genes within cluster 4 illustrated enrichment in several Reactome Pathways, including TGF-β signaling (Figure 10D).

### TGF-β neutralization partially attenuates induction of unique macrophage subsets

**FIGURE 11:**
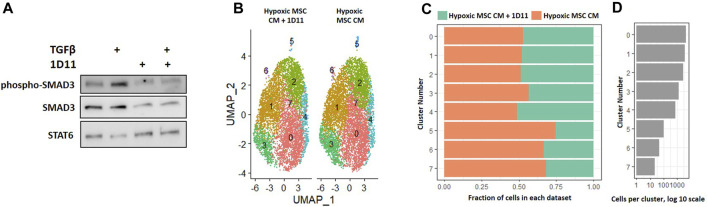
Comparative Analysis between Macrophages Cultured in Hypoxic Medium in the Presence and Absence of the TGF-β Neutralizing Antibody 1D11. **(A)** Macrophages were cultured in hypoxic medium and treated with TGF-β in the presence and absence of the TGF-β neutralizing antibody 1D11 and Western blotting was performed. **(B–D)** scRNA-Seq datasets from macrophages cultured in hypoxic MSC conditioned medium were integrated with macrophages cultured with the addition of the TGF-β neutralizing antibody 1D11 and comparative analyses were performed. **(B)** UMAP representation. The fraction **(C)** and **(D)** cells from each sample within each cluster are shown.

In our comparison analyses, we noted that normoxic CM did not simulate the effects of IL-4, but that hypoxic CM elicited similar clusters in the absence and presence of IL-4. We noted differential regulation of TGF-β signaling when comparing the effects of normoxic CM on macrophage subsets to that of IL-4. Likewise, factors produced by MSCs cultured in hypoxia induced a unique macrophage subset as compared to normoxia (Figure 8). Due to these observations, we next determined if neutralizing any TGF-β isoforms secreted by MSCs into the CM attenuated the effects on macrophage subsets. We first validated that the TGF-β neutralizing antibody 1D11 blocked TGF-β induced signaling (Figure 11A). Next, we compared macrophages cultured in hypoxic MSC CM in the presence and absence of a TGF-β neutralizing antibody. Integration of these two datasets showed mostly even distribution of cells within clusters 0–4; however, a lesser proportion of macrophages cultured in the presence of the TGF-β neutralizing antibody comprised clusters 5–7 (Figures 11B–D). Functional annotation of the differentially expressed genes within clusters 5-7 revealed a role for oxidative phosphorylation, lysosomal function, and metallothioneins, respectively. These results signify that TGF-β partially mediates the effects of MSCs in hypoxic conditions to induce unique macrophage subsets.

### Functional overlap between trophic factors in MSC hypoxic conditioned medium and IL-4

**FIGURE 12:**
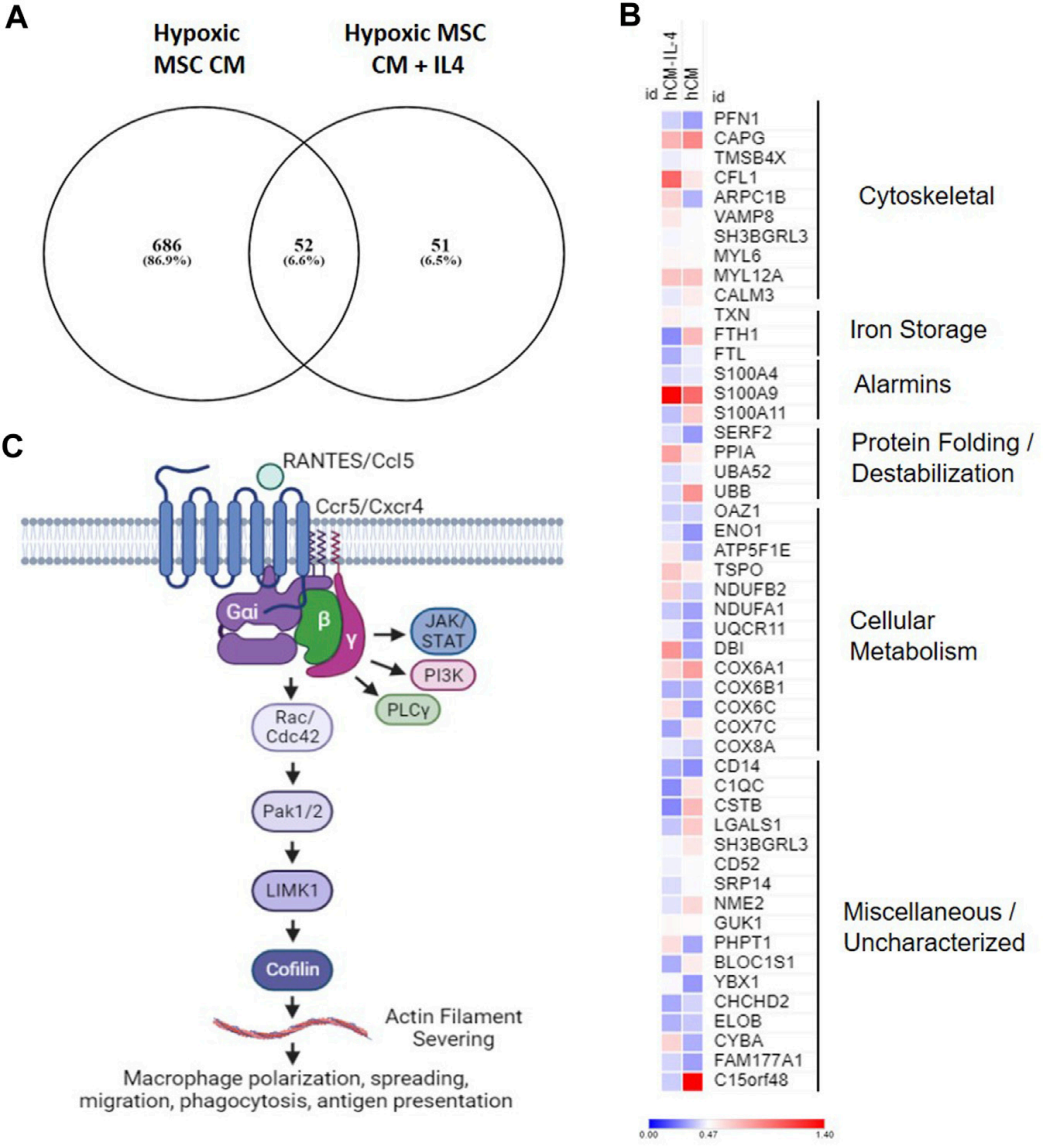
Overlapping Gene Expression. Differentially expressed genes from cluster 4 macrophages treated with hypoxic MSC conditioned medium in the presence and absence of IL-4 were identified. **(A)** Venn diagram. **(B)** Heat map of common genes. **(C)** Overview of putative pathway controling macrophage polarization/function in response to MSC produced factors.

Our data demonstrate that MSCs cultured in hypoxia in the presence and absence of IL-4 produce trophic factors that induce a unique macrophage subset (e.g., cluster 5, Figure 9). To determine the functional overlap of macrophages within cluster 5, we compared differentially expressed genes within this cluster in our hypoxic MSC CM to those identified with the addition of IL-4. Comparison of these two gene sets showed that 686 genes were exclusive to our hypoxic MSC CM dataset, whereas IL-4 treated cells expressed 51 exclusive genes (Figure 12A). Interestingly, these datasets share 55 genes, or 6.6% (Figure 12A). The heatmap shown in Figure 12B depicts the expression data for these common genes. For the most part, shared genes showed similar levels of expression, but cofilin-1, ARPC1B, FTH1, S100A11, UBB, DBI, COX6C, C1QC, CSTB, LAGLS1, PHPT1, BLOC1S1, CYBA and C15orf48 were notable exceptions (Figure 12B). Functional analyses of these common genes showed that cytoskeletal, iron storage, alarmins, protein folding/destabilization, and cellular metabolism genes characterized common genes expressed by cluster five within our analysis.

## Discussion

Intervertebral disc degeneration severity correlates with macrophage numbers within the disc (Nakazawa et al., 2018). MSCs may limit degeneration of the disc in part through their immunomodulatory properties, including effects on macrophages (Teo et al., 2023); however, macrophages constitute a highly plastic and heterogeneous set of cells. Indeed, human degenerative discs contain mixed populations of macrophage subtypes (Nakazawa et al., 2018). In this project, we aimed to define how trophic factors produced by MSCs affect subsets of macrophages.

Hypoxic conditions are often used to simulate the low-oxygen MSCs experience in microenvironments such as bone marrow with minimal effects of MSC survival. Our results contrast with this observation; however, we do note several key differences in our experimental design that may account for this discrepancy. Not only do these cells express markers of hypoxia-induced cell death (e.g., BNIP3), but cell numbers are greatly reduced with increasing time at 2% hypoxia. Our specific cell type under study may have different responses to hypoxia than other types of MSCs. In addition, most studies mimicking low oxygen tension of bone marrow utilize 5% oxygen, whereas we are utilizing 2% to mimic conditions of the IVD.

Our data suggest that multiple subtypes of macrophages exist. When looking at types of macrophage subsets identified in our analyses, we noted several functional annotations that defined clusters, including categories such as antigen presentation, alarmins, apolipoproteins, and chemokines. Antigen presenting macrophages are the most prominent in inflammatory sites and specialized for clearing necrotic and apoptotic material, so some macrophage subsets may specialize in these functions. Likewise, other macrophage subtypes may more potently recruit other cell types through enhanced chemokine production. In contrast, apolipoprotein signaling converts macrophages to an anti-inflammatory phenotype (Baitsch et al., 2011; Zheng et al., 2018). Defining how these different classes of macrophages function during intervertebral disc degeneration and/or how MSCs placed within the disc modulate macrophage subsets by would likewise help to define mechanisms of action.

Our combined data showing enrichment of chemokine signaling, Rho GTPase Effectors, and G alpha (i) signaling point to a role for Ccl5/Ccr5 signaling and regulation of macrophage spreading, migration and induced inflammation regulated by the actin severing protein cofilin (Figure 12C). These data suggest that MSCs placed in the hypoxic environment of the disc may induce this pathway by macrophages leading to the generation of a unique macrophage subset similar to that induced by IL-4. Chemokine signaling plays a role in intervertebral disc degeneration (Hu et al., 2020). Nucleus pulposus cells within the IVD produce chemokines such as Ccl2, 5, 7, and 8 that promote ingress of macrophages to the IVD (Liu et al., 2017; Hu et al., 2020). Likewise, chemokines levels correlate with disease severity (Hiyama et al., 2022); however, it is unclear if increased Ccl5 levels are a cause or consequence of degeneration. In this study, we demonstrate that secreted factors produced by MSCs in hypoxic environments induce expression of Ccl5/Ccr5 by a specific subset of macrophages, and that these factors overlap with those induced by IL-4. Activation of Ccr5-dependent signaling induces macrophage survival and ECM remodeling (Di Marzio et al., 2005). Moreover, activation of Ccr5-dependent signaling in macrophages alters actin dynamics to facilitate lamellipodia formation, migration, and invasion (Di Marzio et al., 2005; Tyner et al., 2005). Within tumor environments, Ccr5 signaling induces immunosuppressive macrophage phenotypes (Halama et al., 2016). Given our data demonstrating common expression of genes with this pathway (Ccl5, Ccr5, Cfl1) that overlap with those induced by IL-4, mechanistic studies aimed at understanding the role of Ccl5/Ccr5 signaling within macrophages, the specific functions of this macrophage subset, and the downstream effects on disc degeneration are warranted.

When comparing the effects of hypoxic MSC CM in the presence and absence of IL-4, we noted one key difference in gene expression. We found that IL-4 was required to induce genes (e.g., Ccl3, IL-1β, Ccl4, Cxcl8, Timp1, and ICAM1) related to IL-10 signaling. IL-10 acts as an anti-inflammatory cytokine that can limit production of reactive oxygen species and promote metabolic reprogramming (Ip et al., 2017); thus, IL-10 may limit disc degeneration through effects on endogenous macrophages (Ge et al., 2020). IL-4 induces the production of IL-10 in other cell types (Mitchell et al., 2017), which may explain the difference between these two cell populations.

Our data point to a role for TGF-β signaling as an MSC-derived factor affecting macrophage subsets. We first demonstrate that hypoxia induces production of a trophic factor(s) produced by MSCs that mimics the effects of the anti-inflammatory cytokine IL-4. The unique macrophage subset induced by hypoxic CM in both the presence and absence of IL-4 demonstrates this point; however, MSCs cultured at atmospheric oxygen tension did not induce an analogous unique macrophage subset to that of hypoxic CM in the presence or absence of IL-4. Comparative analyses between macrophages cultured in normoxic CM and those cultured in hypoxic CM plus IL-4 implicated altered TGF-β signaling. Likewise, neutralization of TGF-β within the CM of hypoxic cultured MSCs partially blocked the effects on macrophage responses. TGF-β signaling has a complex role within the intervertebral disc. TGF-β signaling facilitates proper development of the intervertebral disc (Baffi et al., 2004; Baffi et al., 2006; Colombier et al., 2014; Ashley et al., 2016; Sivakamasundari et al., 2017); thus, it is not surprising that TGF-β likewise has reported roles in promoting intervertebral disc regeneration (Matta et al., 2018; Bello et al., 2021). In contrast, other studies have demonstrated that aberrant TGF-β signaling can lead to disc degeneration (Bian et al., 2016; Zieba et al., 2016). Similarly, TGF-β limits inflammatory responses of macrophages in other cell contexts (Gong et al., 2012; Zhang et al., 2016). Our study suggests that TGF-β may control macrophage phenotypes; thus, understanding the how TGF-β modulates macrophage activities within a model of IVD degeneration is of future interest.

Specific subsets of macrophages may be key to limiting inflammation observed during IVD degeneration, as the effects of broad macrophage depletion have contrasting results. For instance, broad clearance of all macrophages *in vivo* via clodronate liposomes limited production of inflammatory cytokines when CD11B^+^ cells from the intervertebral disc are cultured *in vitro* (Miyagi et al., 2018). Similarly, reduced macrophage infiltration and inflammatory mediators characterize the enhanced healing of superhealer MRL/MpJ mice (Lewis et al., 2013). Likewise, depletion of macrophages in models of collagen-induced arthritis reduced levels of inflammatory cytokines (Li et al., 2012). In contrast, transient genetic macrophage depletion in a murine model of disc degeneration lead to enhanced numbers of neutrophils and B cells within the disc, but did not alter levels of inflammatory markers (Xiao et al., 2023). This was also observed in pre-clinical models of obesity-associated osteoarthritis, where short-term genetic depletion of macrophages enhanced inflammation and increased levels of other immune cell types within the joint (Wu et al., 2017). Given these contrasting effects of broad macrophage depletion, there is an urgent need to define specific subsets of macrophages that limit local inflammation and/or reduce leukocyte infiltration to the intervertebral disc during degeneration. Moreover, understanding how MSCs modulate macrophage subsets may be key to defining mechanisms of action and designing effective modes of therapeutic use.

In this study, we sought to identify basic mechanisms underlying MSC-mediated modulation of macrophage phenotypes, but our work has several key limitations. First, our data are limited to the effects on human macrophages cultured *in vitro* and may not be reflective of macrophage subsets within the degenerative intervertebral disc. Though out of the scope of our study, an analysis of macrophage subsets within normal and degenerative discs would be beneficial, as well as functional validation of our single cell data to determine how MSC delivery to the disc affects macrophage subsets. Moreover, the results of single-cell analysis could be further validated by sorting for cell surface makers that define unique macrophage subsets and determining how these subsets impact disc degeneration and disc cell health either *in vivo* or *in vitro*. We also utilized human bone marrow as our source of macrophages; thus, this source would be reflective of macrophages recruited to this disc from the periphery, but may not mimic tissue resident macrophages within the IVD that are likely embryonically derived (Koroth et al., 2023). Moreover, detailed future studies examining the effects of MSCs on macrophage phenotypes during the course of intervertebral disc degeneration would directly validate impacts on disease progression. In our comparison analyses, we noted that normoxic CM did not simulate the effects of IL-4, but that hypoxic CM elicited similar clusters in the absence and presence of IL-4; however, these data do not identify the effects of IL-4 alone on macrophage subsets. Likewise, our finding that MSCs require TGF-β to elicit anti-inflammatory responses from macrophages should be validated in preclinical models. Our data also show that TGF-β neutralization partially limited the effects; therefore, MSCs may require other factors fully to limit disc degeneration. Our experimental design also only tested the effects of trophic (e.g., secreted) factors, but membrane-bound or tethered factors may likewise mediate the anabolic effects of MSCs.

Overall, our data suggest that MSCs produce trophic factors that induce a unique macrophage subset approximating the effects of the anti-inflammatory cytokine IL-4. This effect is hypoxia-dependent, as CM derived from MSCs cultured in normoxic conditions did not mimic the effects of IL-4. Moreover, our results partially attributed these effects to TGF-β signaling.

## Data Availability

Sequencing data are deposited into the Gene Expression Omnibus (GEO accession numbers GSE246842, GSM7879984, GSM7879985, GSM7879986, GSM7879987) and all other data are contained within this manuscript.
